# Assessing the therapeutic impact of resveratrol in ALS SOD1-G93A mice with electrical impedance myography

**DOI:** 10.3389/fneur.2022.1059743

**Published:** 2022-12-22

**Authors:** Janice A. Nagy, Carson Semple, PuiChi Lo, Seward B. Rutkove

**Affiliations:** Department of Neurology, Beth Israel Deaconess Medical Center, Harvard Medical School, Boston, MA, United States

**Keywords:** electrical impedance myography, resveratrol, amyotrophic lateral sclerosis, mouse, therapy

## Abstract

To aid in the identification of new treatments for amyotrophic lateral sclerosis (ALS), convenient biomarkers are needed to effectively and uniformly measure drug efficacy. To this end, we assessed the effects of the nutraceutical resveratrol (RSV) on disease onset and overall survival in SOD1-G93A (ALS) mice and compared several standard biomarkers including body mass, motor score (MS), paw grip endurance (PGE), and compound motor action potential (CMAP) amplitude, with the technique of electrical impedance myography (EIM) to follow disease progression. Eighteen ALS mice (nine females, nine males) received RSV in the chow (dose: 120 mg/kg/day) starting at 8 weeks of age; 19 ALS mice (nine females, 10 males) received normal chow; 10 wild type (WT) littermates (five females, five males) fed standard chow served as controls. Biomarker assessments were performed weekly beginning at 8 weeks. No differences in either disease onset or overall survival were found between RSV-treated and untreated ALS mice of either sex; moreover, all biomarkers failed to identify any beneficial effect of RSV when administered at this dose. Therefore, for the comparative evaluation of the ability of the various biomarkers to detect the earliest symptoms of disease, data from all animals (i.e., RSV-treated and untreated ALS mice of both sexes) were combined. Of the biomarkers tested, EIM impedance values, i.e., surface EIM longitudinal phase at 50 kHz (LP 50 kHz), and CMAP amplitude showed the earliest significant changes from baseline. LP 50 kHz values showed a rate of decline equivalent to that of CMAP amplitude and correlated with both PGE and CMAP amplitude [Spearman rho = 0.806 (*p* = 0.004) and 0.627 (*p* = 0.044), respectively]. Consistent with previous work, these findings indicate that surface EIM can serve as an effective non-invasive biomarker for preclinical drug testing in rodent models of ALS.

## Introduction

Amyotrophic lateral sclerosis (ALS), is a progressive neurodegenerative disease that affects nerve cells in the brain and the spinal cord leading to loss of upper and lower motor neurons, muscle denervation, loss of voluntary muscle control, muscle atrophy, and eventual death. In order to assess new potential drug therapies, most preclinical studies using ALS rodent models rely on a variety of measures such as body mass, survival, muscle girth, electrophysiological testing, and assorted behavioral parameters including paw grip endurance test (PGE), rotarod, treadmill, open field activity, and/or motor score (MS) (1–11). To aid in the identification of new treatments for ALS, additional biomarkers are needed to more effectively and uniformly measure disease onset and progression.

Electrical impedance myography (EIM) has shown promise as a biomarker in both human and animal studies of ALS (12, 13). In surface EIM, a rigid 4-electrode array is placed on the skin overlying a muscle of interest, and a weak, multi-frequency (1 kHz−1 MHz) electrical current is passed across the two outer electrodes and into the muscle. The two inner electrodes record the impedance to current flow by the muscle fibers (14–17). Alterations in the measured impedance values (i.e., resistance, reactance) and the derived phase values provide data on muscle condition such as myofiber size and the development of fat and connective tissue as occurs in longer standing disease (18–20).

Human studies have shown that surface EIM longitudinal phase at 50 kHz (LP 50 kHz) is sensitive to decline in ALS patients and the rate of deterioration correlates to length of survival, suggesting that surface EIM could potentially serve as a surrogate outcome measure (12). Moreover, when surface EIM was used to track a single rapidly deteriorating muscle, excellent sensitivity to overall ALS progression was achieved, and 81% fewer subjects would be required to determine clinical benefit as compared to standard measures such as ALSFRS-R (21). Additional advantages of surface EIM include that it is non-invasive, painless, rapid to apply, requires minimal training, is highly reproducible, and can be used to study virtually any superficial muscle (22, 23).

In preclinical studies, surface EIM applied to the SOD1-G93A rat model of ALS demonstrated sensitivity to disease progression and a strong relationship between the rate of decline in LP 50 kHz values and length of survival of the animals (13). In a study designed to evaluate drug efficacy, although SOD1-G93A ALS mice treated with riluzole showed no increase in time of disease onset or overall survival (24), there was good correlation between LP 50 kHz values and other biomarkers including paw grip endurance (PGE), and Compound Muscle Action Potential (CMAP) amplitude, supporting the conclusion that changes in surface EIM values reflect motor neuron loss and declining motor function.

Hoping to further assess EIM's sensitivity to drug effect, we undertook an additional study, focusing on the potential therapeutic effect of the nutraceutical resveratrol (3,4′,5- trihydroxystilbene) (RSV). RSV, a polyphenol compound found in the skin of red grapes, has been shown to mimic effects of caloric restriction, exert anti-inflammatory and anti-oxidative effects, and affect the initiation and progression of different diseases through a variety of mechanisms (25). We recently demonstrated the ability of RSV to mitigate muscle deconditioning in a rat model of reduced mechanical loading (26). RSV has also received considerable attention for its potential neuroprotective effects in some neurodegenerative disorders (27), but there is conflicting information about its value in the treatment of ALS (28). One study reported that a single dose of RSV at 25 mg/kg in the SOD1-G93A ALS mouse model did not improve motor abilities or extend survival in ALS mice (29). A second study used intraperitoneal injections of RSV at a dose of 20 mg/kg/twice a week, which were reported to improve survival and delay disease onset in this model (30). A similar positive effect of RSV on survival and motor function was observed with a higher dose of RSV (160 mg/kg/day) administered orally in ALS mice (31).

Based on these previously published papers, we evaluated longitudinally a group of SOD1-G93A ALS mice treated with RSV vs. a group of untreated animals with two goals in mind: (1) to assess the effects of RSV treatment on disease progression and overall survival; and (2) to compare the effectiveness of several biomarkers [i.e., CMAP amplitude, paw grip endurance (PGE), motor score (MS), and surface EIM] to detect disease onset and progression, as well as the potential drug efficacy of RSV in this model.

## Materials and methods

### Animals

All experimental procedures were approved by the Institutional Animal Care and Use Committee (IACUC) at Beth Israel Deaconess Medical Center (Protocol Number 031-2018) and performed in accordance with The Guide for the Care and Use of Laboratory Animal, 8th edition, 2011 of the National Institutes of Health. Breeding colonies of ALS SOD1-G93A (ALS) mice were established from animals obtained from Jackson Laboratories (Bar Harbor, Maine) [four males B6SJL-Tg(SOD1-G93A)1Gur/J, hemizygous for Tg(SOD1^*^G93A)1Gur, strain #002726 and 12 B6SJLF1/J Females, strain #100012]. Pups were genotyped by tail snip and weaned at 21 days. A total of 37 ALS mice were studied (18 females and 19 males). In creating the treatment and no treatment groups, we combined mice from 11 different litters, but did not have significant numbers to litter match the treatment vs. the non-treatment groups. In addition, WT littermates (five females, five males) served as controls. Animals were housed in micro-isolator cages in groups of up to five mice/cage in the animal facility equipped with a 12:12 light/dark standard lighting cycle, and were monitored on a daily basis by research staff, evaluating conditions of fur, grooming, the presence of porphyrin staining, mobility and gait, and ability to feed.

### Treatment with resveratrol

HPLC-purified trans-resveratrol (RSV), (obtained from Megaresveratrol.net, Candlewood Stars, Inc, Danbury, CT, United States) was incorporated into Purina Formulab Diet 5008 at a concentration of 0.1% by Envigo Teklad Diets, Madison WI. A cohort of untreated ALS mice was fed normal chow (NC), while another cohort of ALS mice was fed RSV incorporated in their chow. Starting at 8 weeks of age, 18 ALS animals (nine females, nine males) were fed with RSV chow ad lib; 19 ALS mice (nine females, 10 males) received Purina Formulab Diet 5008. WT littermates (five females, five males) were fed standard chow and served as controls. All animals in the study were switched to Gel-packs (DietGelH 76A, PharmaSer, Framingham, MA) when the first ALS mice in either treatment group became too weak to reach their chow. These Diet Gel-packs did not contain Resveratrol. All animals were then followed out to the anticipated time of death (~120–140 days).

### Dosage of resveratrol

RSV-treated ALS mice were fed Purina Formulab Diet 5008 chow that had been incorporated with RSV at an initial concentration of at a concentration of 0.1%. An analysis of the chow immediately prior to initiation of the study (after a delay of 6 months due to lab shutdown caused by the COVID-19 pandemic) yielded an RSV concentration of 0.075%. Therefore, based on an average daily food intake of 3–5 g per 25 g body mass, RSV was administered to the ALS mice at an average dose of ~120 mg/kg/day (i.e., 25% less than our originally planned target of 160 mg/kg/day).

### Behavioral measurements

ALS mice were monitored daily to assess feeding and movement throughout the course of disease. Since the BIDMC IACUC would not approve the commonly used endpoint in ALS studies, i.e., a prolonged (>30 s) righting reflex (7), an alternative approach was approved and used for all animals. A standard motor score (MS) assessment (graded on a 0–4 scale) was assigned to all animals (4, 24). A MS of four was given for animals with no sign of motor dysfunction; three for animals with detectable tremors when suspended by the tail; two when the animals had mild difficulty ambulating; one when mice were dragging at least one of their hind limbs; zero when both hind limbs were fully paralyzed. Disease onset was determined when an animal received a MS equal to 3. When their MS reached a value of 0, mice were deemed moribund and euthanized by inhalation of carbon dioxide (CO_2_) gas delivered from a compressed gas canister.

The paw grip endurance test (PGE) (4, 24), also known as the hanging wire or inverted screen test (1–4), was performed bi-weekly beginning at 8 weeks of age to assess muscle strength (32). Briefly, each mouse was placed in the center of a 30 × 42 cm wire rack with 1 × 1 cm square openings and 1 mm diameter wire. The wire rack was inverted quickly and placed on a 25 × 37 cm rectangular Plexiglas support 25 cm above a padded surface. The time until the mouse let go with all four limbs and dropped onto the padded surface below was noted. Each mouse was given three attempts to hold onto the inverted lid for an arbitrary maximum time of 120 s and the longest time achieved by each mouse was recorded.

### Compound muscle action potential amplitude

Compound muscle action potential (CMAP) amplitudes were measured bi-weekly using the Natus UltraPro S100 EMG system with Synergy software (Natus Neuro, Middleton WI) as previously described (33).

### Electrical impedance myography

All electrical impedance myography (EIM) measurements were made bi-weekly with the mView impedance spectroscopy system (Myolex Inc., Boston, MA) using 41 logarithmically spaced frequencies from 1 kHz to 10 MHz. Surface EIM measurements were performed as described (34) with the animals under 1% isoflurane anesthesia with a heating pad underneath the animal to maintain consistent body temperature at 37°C. After the fur was clipped, a depilatory agent was applied for 1 min and the skin cleaned with 0.9% saline solution. This process was repeated a total of three times to ensure complete fur removal. The leg was taped to the measuring surface at ~45° angle extending out from the body. A fixed rigid four-electrode array was applied over the gastrocnemius (GA) muscle to obtain longitudinal measurements (35). Measurements were repeated twice and averaged. The array was rotated 90 degrees, and measurements repeated to obtain transverse values. Longitudinal and transverse resistance (*R*) and reactance (*X*) values were collected across the entire frequency range and longitudinal and transverse phase values were calculated as arctan (*X*/*R*), respectively. Longitudinal phase values at 50 kHz (LP 50 kHz) were used in subsequent biomarker comparisons.

### Statistical analyses

Biomarker data are reported as either the mean ± standard error across the groups. Kaplan-Meier curves were constructed to assess the effect of RSV on the time of disease onset (determined as the time of the first recorded MS value = 3) and length of overall survival (determined as the time of euthanasia required when the MS value = 0). The average time of disease onset and average survival times were reported as mean ± SEM. The median time of disease onset and the median survival time were reported together with their respective Hazard ratios (Log-rank) and their 95% confidence intervals. Basic statistical analyses of the physiological and impedance values were performed using GraphPad Prism V8 (GraphPad Software, Inc. La Jolla, CA). Unpaired *t*-tests were used to compare the means between two groups; multiple group comparisons were performed by one-way ANOVA with Tukey's multiple comparisons test. The rates of decline of the various biomarkers were determined by simple linear regression. The linearity of the rate of decline of the various biomarkers was quantified as described (24) by performing a least-squares fit based on the average data for each week across the entire measurement period. Residuals for each of the weeks were calculated; the absolute value of these residuals were taken and averaged for each measure and then divided by the average value of that parameter across all measurement points, thus providing a gauge of how data from each week deviated from the regression line. Spearman's correlation coefficient, rho, was calculated to assess the relationship between survival and rate of decline in various biomarker values, as well as for correlations between LP 50 kHz and PGE and CMAP amplitude. For all analyses, *p* < 0.05, two-tailed was considered as significant.

## Results

### Effect of resveratrol on time of disease onset and animal survival

**Figure 1:**
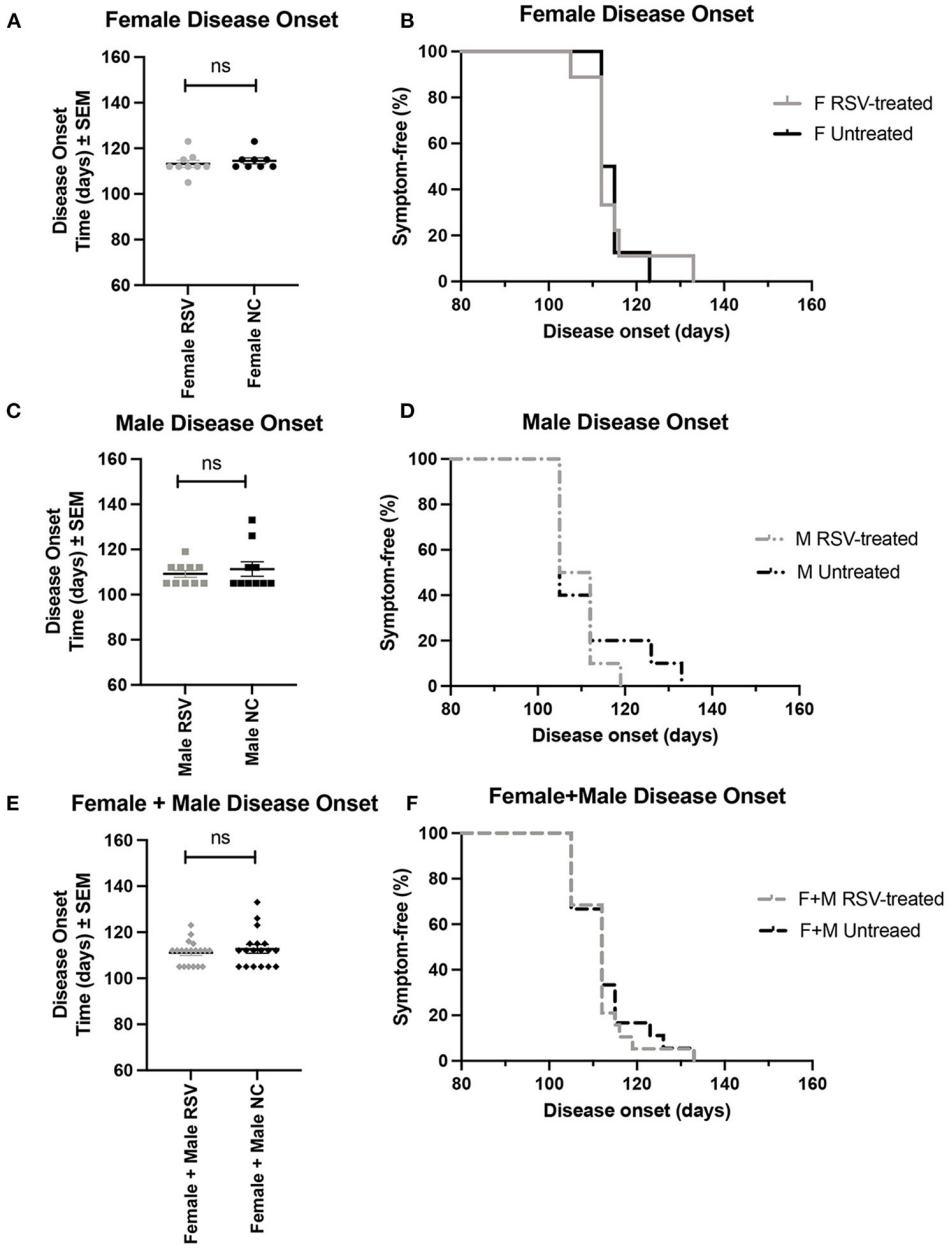
Scatter plots and Kaplan-Meier curves showing disease onset for female, male, and combined (female + male) RSV-treated vs. untreated ALS mice. Disease onset was determined as the time of the first recorded MS = 3. **(A)** Female disease onset scatter plot. **(B)** Female disease onset Kaplan-Meier plot. **(C)** Male disease onset scatter plot. **(D)** Male disease onset Kaplan-Meier plot. **(E)** (Female + male) disease onset scatter plot. **(F)** (Female + male) disease onset Kaplan-Meier plot. RSV, Resveratrol-treated animals; NC, Untreated animals received normal chow. Data in panels **(A, C, E)** are expressed as mean ± SEM. ns, non-significant.

**Figure 2:**
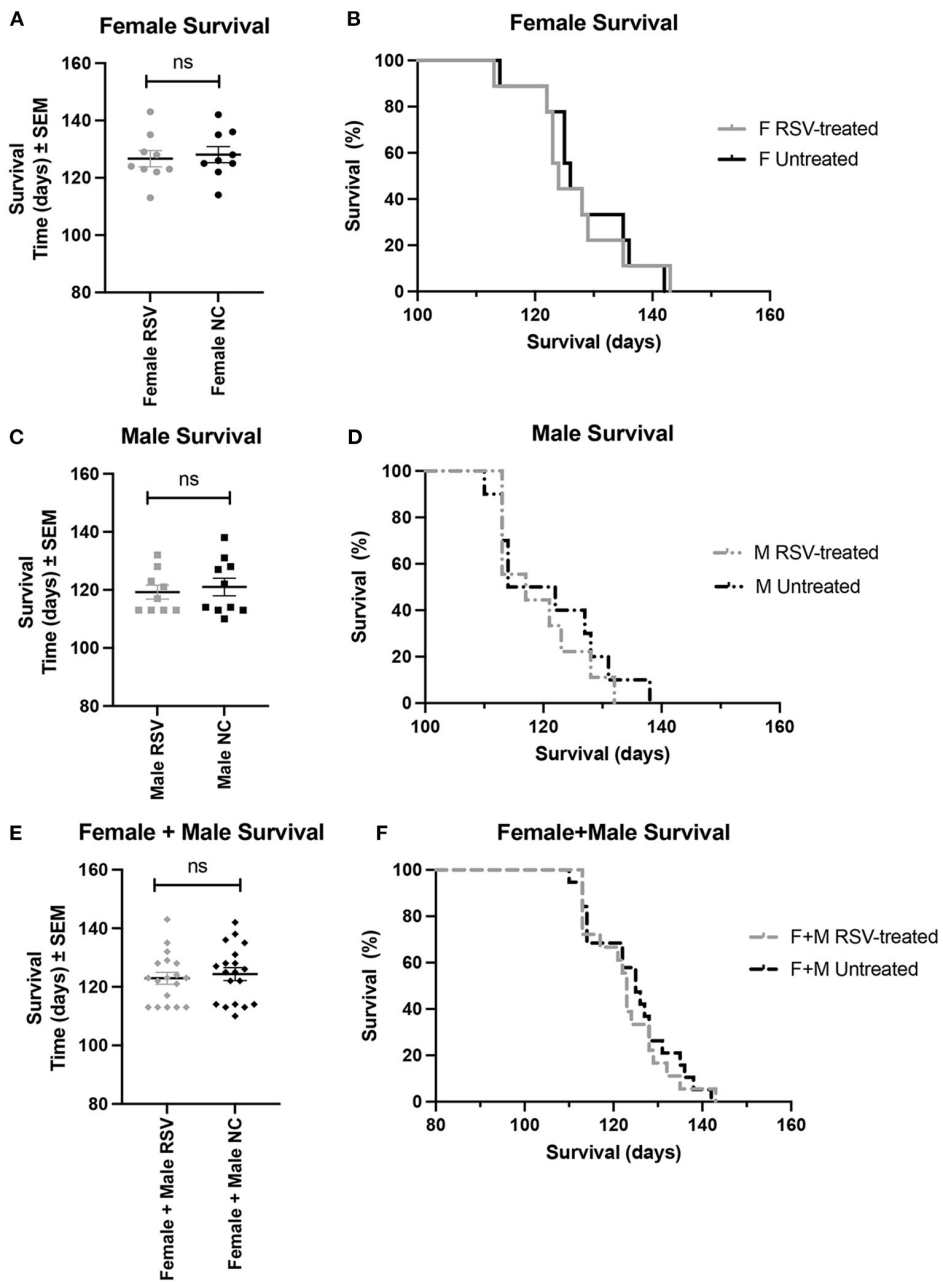
Scatter plots and Kaplan-Meier curves showing survival for female, male, and combined (female + male) RSV-treated vs. untreated ALS mice. Survival time was determined as the time when the MS = 0 necessitating required euthanasia. **(A)** Female survival time scatter plot. **(B)** Female survival time Kaplan-Meier plot. **(C)** Male survival time scatter plot. **(D)** Male survival time Kaplan-Meier plot. **(E)** (Female + male) survival time scatter plot. **(F)** (Female + male) survival Kaplan-Meier plot. RSV, Resveratrol-treated animals; NC, Untreated animal received normal chow. Data in panels **(A, C, E)** are expressed as mean ± SEM. ns, non-significant.

Figures 1, 2 show the scatter plots and the Kaplan-Meier curves for time of disease onset (Figure 1), determined as the time of the animal's first recorded MS = 3, and overall survival (Figure 2), determined as the time of required euthanasia when an animal's MS = 0, for both RSV-treated and untreated ALS animals fed normal chow (NC), separated by sex, as well as when the sexes were combined. As shown in Figures 1, 2 there was no significant difference in the average time of disease onset or average length of survival between the RSV-treated and the untreated ALS animals for either sex when studied individually or when the sexes were combined. As shown in Table 1, neither the median time of disease onset nor the median survival time differed between the RSV-treated and the untreated ALS animals for either sex when studied individually or when the sexes were combined.

**Table 1:** Median disease onset and median survival of RSV-treated and untreated ALS mice.

**Group**	**Median disease onset (days)** ^a^				**Median survival (days)** ^b^			
	**RSV-treated**	**Untreated**	***p*** **-value** ^c^	**Hazard ratio** ^d^	**RSV-treated**	**Untreated**	***p*** **-value** ^c^	**Hazard ratio** ^d^
Female ALS	112.0	113.5	0.953	1.021 (0.394–2.646)	124.0	126.0	0.875	1.071 (0.425–2.699
Male ALS	108.5	105.0	0.563	1.182 (0.490–2.848)	117.0	118.0	0.615	1.232 (0.496–3.061)
Female + male ALS	112.0	112.0	0.675	1.109 (0.582–2.112)	123.0	125.0	0.691	1.129 (0.591–2.156)

a Determined as first time the motor score = 3.

b Determined as the time when motor score = 0, necessitating euthanasia.

c *p*-value calculated using the Log-rank (Mantel-Cox test).

d Hazard ratio (log-rank) and 95% CI of ratio.

**Figure 3:**
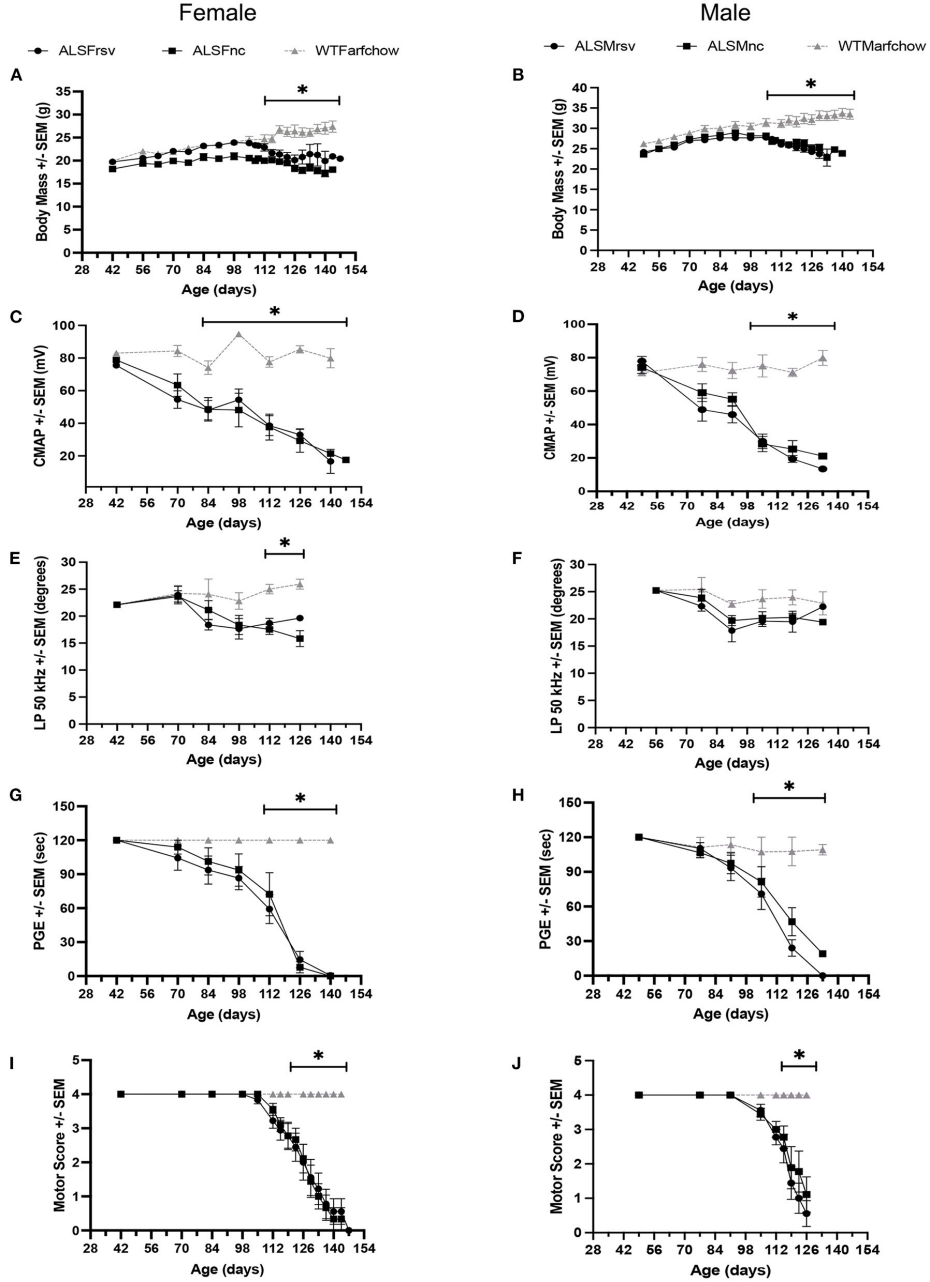
Bi-weekly measures for RSV-treated vs. untreated female and male ALS mice vs. WT animals. **(A, B)** Body mass. **(C, D)** CMAP amplitude. **(E, F)** LP 50 kHz. **(G, H)** PGE. **(I, J)** Motor score. ALSF, ALS female; ALSM ALS male; WTF, wild type female; WTM, wild type male. RSV, Resveratrol-treated animals; NC, Untreated animal received normal chow. Significant difference from WT values for that measure are indicated by “*”.

As shown is Figure 3 for both female and male ALS mice, the rates of decline for body mass, CMAP amplitude, LP 50 kHz, PGE, and MS, were similar between untreated and RSV-treated animals. Body mass did not show a significant change from WT until 112 days for females and slightly earlier at 105 days for males. Similarly, the decline in MS became significant at 119 days for both females and males, while the change in CMAP amplitude became significant at 84 days for females and 105 days for males. The decline in PGE became significant at 112 days in females and at 105 days in males. The decline in LP 50 kHz occurred between 84 and 98 days of age for both female and male ALS mice and was significantly different from the WT in females at day 112. Table 2 shows the individual slopes of decline over time for each biomarker. The *p*-values indicate that there were no significant differences in the rates of decline of any biomarker between RSV-treated and untreated ALS mice for either sex.

**Table 2:** Rates of change for body mass, CMAP amplitude, LP 50 kHz, PGE, and motor score in RSV-treated vs. untreated ALS animals.

	**Female ALS**			**Male ALS**		
	**RSV-treated**	**Untreated**	***p*** **-value** ^a^	**RSV-treated**	**Untreated**	***p*** **-value** ^a^
Body mass (g/week)	−0.704 ± 0.172	−0.641 ± 0.111	0.765	−0.956 ± 0.125	−0.759 ± 0.148	0.336
CMAP (mV/week)	−3.429 ± 0.533	−4.080 ± 0.681	0.455	−5.659 ± 0.637	−5.128 ± 0.618	0.551
EIM LP 50 kHz (degrees/week)	−0.3630 ± 0.112	−0.501 ± 0.105	0.3702	−0.7625 ± 0.153	−0.6858 ± 0.127	0.6994
PGE (s/week)	−5.628 ± 1.324	−4.571 ± 1.551	0.651	−8.513 ± 1.261	−6.424 ± 1.125	0.222
Motor score (points/week)	−0.652 ± 0.068	−0.750 ± 0.059	0.275	−0.714 ± 0.052	−0.607 ± 0.069	0.215

a *p*-values compare rates of change for RSV-treated and untreated groups to determine if rates of change are different from each other.

### Alterations in biomarker values over time

**Figure 4:**
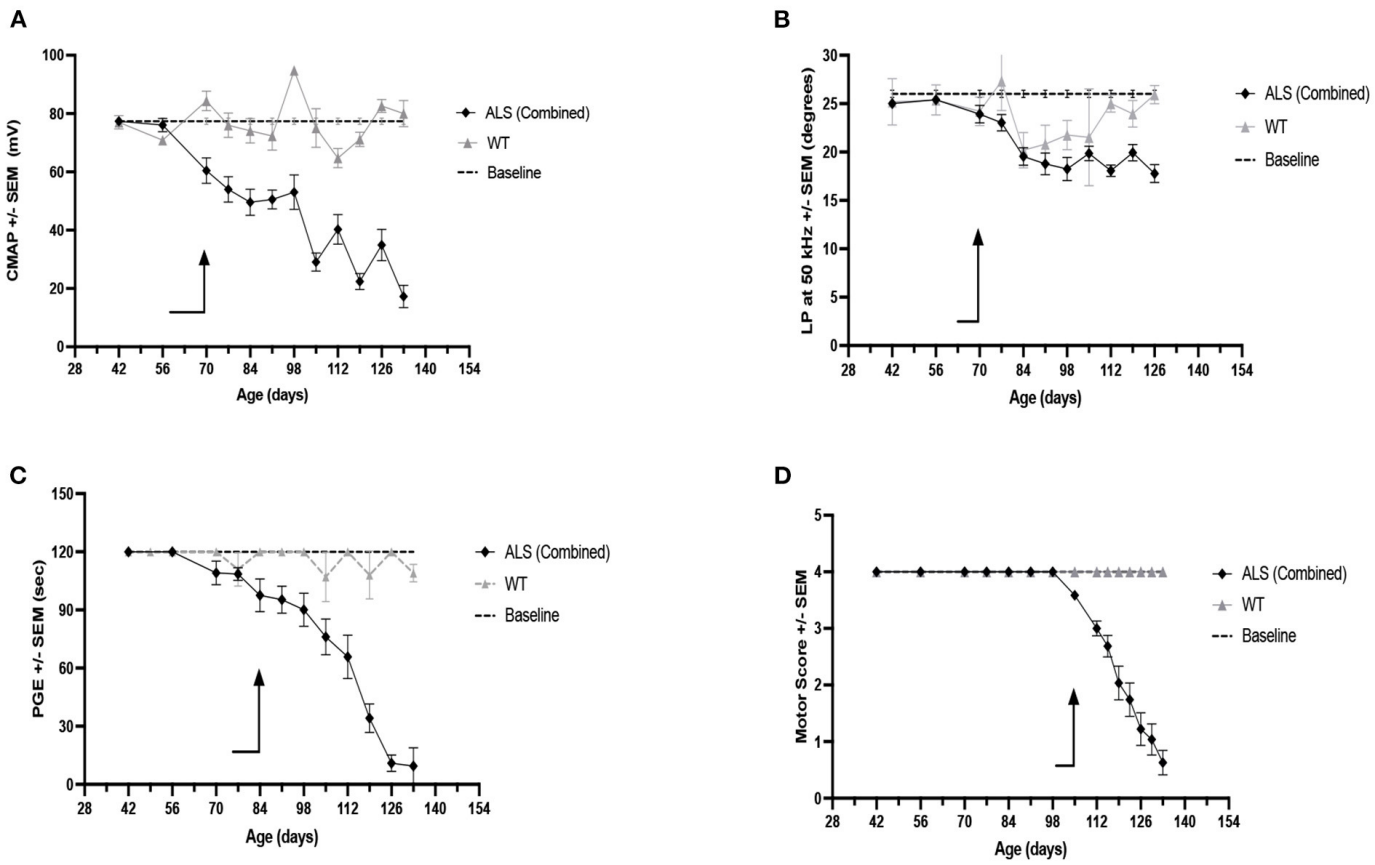
Weekly measures for Combined RSV-treated (F + M) and untreated (F + M) ALS mice vs. combined WT (F + M) biomarker values and vs. each biomarker baseline values. **(A)** CMAP amplitude. **(B)** LP 50 kHz. **(C)** PGE. **(D)** Motor score. Arrows indicate time of first consistent significant difference from baseline values for that measure.

Since neither female nor male RSV-treated animals showed a difference from their untreated counterparts in any of the physiological or electrophysiological biomarkers examined, the data from all animals i.e., the RSV-treated and untreated ALS animals of both sexes, were combined to increase statistical power. A comparison of CMAP amplitude, LP 50 kHz, PGE, and MS values is presented in Figure 4. The arrows in each figure mark the time of significant change from their baseline values (*p* < 0.05) for two consecutive weeks or longer; this information is summarized in Table 3. When all of the experimental data was combined, the LP 50 kHz values showed a similar change from baseline as that of CMAP amplitude, i.e., at 10 weeks (i.e., 70 days), and slightly better than PGE, i.e., at 12 weeks (i.e., 84 days), whereas the MS did not show a significant change until 15 weeks (i.e., 105 days). The rates of decline, and the linearity of the decline, for each biomarker are summarized in Table 3. As can be seen in Table 3, LP 50 kHz had the smallest mean residual, indicating that the surface EIM impedance data would track very closely along a fitted line. Compared to CMAP amplitude, which also became significantly different from baseline at 10 weeks of age, and which has the next lowest average residual value, the LP 50 kHz residuals were significantly lower (*p* < 0.0001). In comparison, PGE showed a significant change from baseline at 12 weeks and MS showed a significant change at 16 weeks and these two biomarkers had mean residuals of 0.167 and 0.201 respectfully.

**Table 3:** Rates of change for CMAP amplitude, LP 50 kHz, PGE, and motor score for combined [RSV-treated (F + M) + untreated (F + M)] vs. baseline.

**Biomarker**	**All data combined [RSV-treated (F + M) + untreated (F + M)] vs. baseline**	***p*** **-value^a^**	**Normalized mean absolute residual from fitted line**	**Time of disease onset (weeks) vs. baseline**
CMAP (mV/week)	−4.305 ± 0.361	*p* < 0.0001	0.113	10
LP 50 kHz (degrees/week)	−0.789 ± 0.100	*p* < 0.0001	0.058	10
PGE (s/week)	−6.496 ± 1.022	*p* < 0.0001	0.167	12
Motor score (points/week)	−0.723 ± 0.051	*p* < 0.0001	0.201	16

a *p*-values compare rates of change for the combined [RSV-treated (F + M) + untreated (F + M)] groups to determine if different from baseline.

### Correlations between survival and rate of deterioration of LP 50 kHz, PGE, CMAP amplitude, and motor score

**Figure 5:**
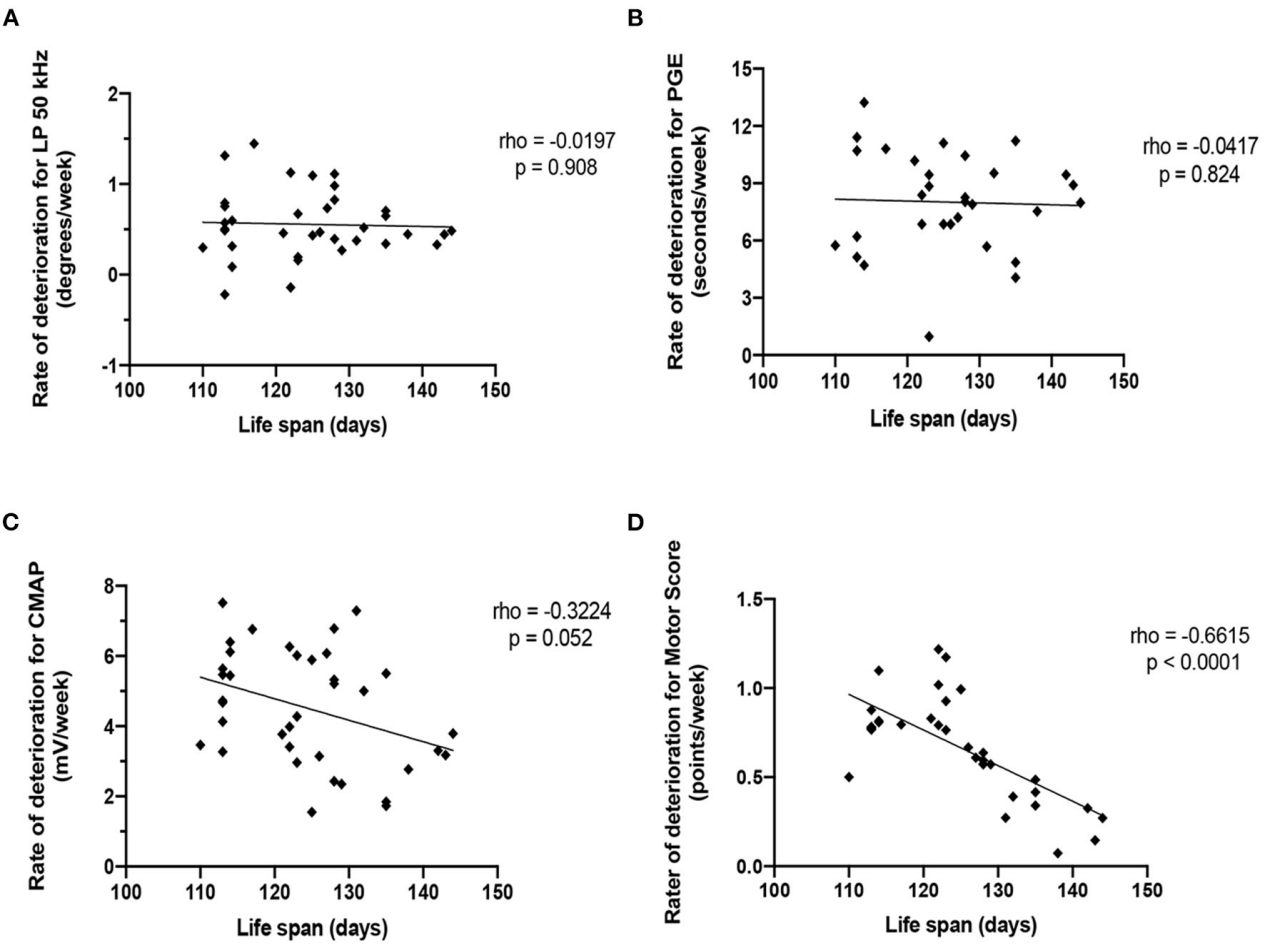
Correlation of the rate of deterioration of each biomarker with life span. **(A)** LP 50 kHz. **(B)** PGE. **(C)** CMAP amplitude. **(D)** Motor score.

Figure 5 shows the correlation between the mean rate of deterioration of LP 50 kHz, PGE, CMAP amplitude and MS with survival for that animal. Neither the rate of deterioration of LP 50 kHz nor the rate of deterioration of PGE significantly correlated with lifespan (rho: −0.0197, *p* = 0.908 and rho: −0.0417, *p* = 0.824, respectfully). CMAP amplitude correlated with lifespan only slightly but not significantly better with correlation coefficients of rho: −0.3224, *p* = 0.052. Meanwhile, the rate of MS deterioration demonstrated significant correlations (rho: −0.6615, *p* < 0.0001). It is not surprising the rate of deterioration of MS had the best correlation with lifespan since the MS value was used to determine the time of mandatory euthanasia. Importantly, as shown in Figure 2, the variation in lifespan among the animals is relatively small (range: 113–143 days for combined sexes); therefore, finding significant correlations between the rate of disease progression of other biomarkers and overall survival remain challenging.

### Correlations between LP 50 kHz and PGE and CMAP

**Figure 6:**
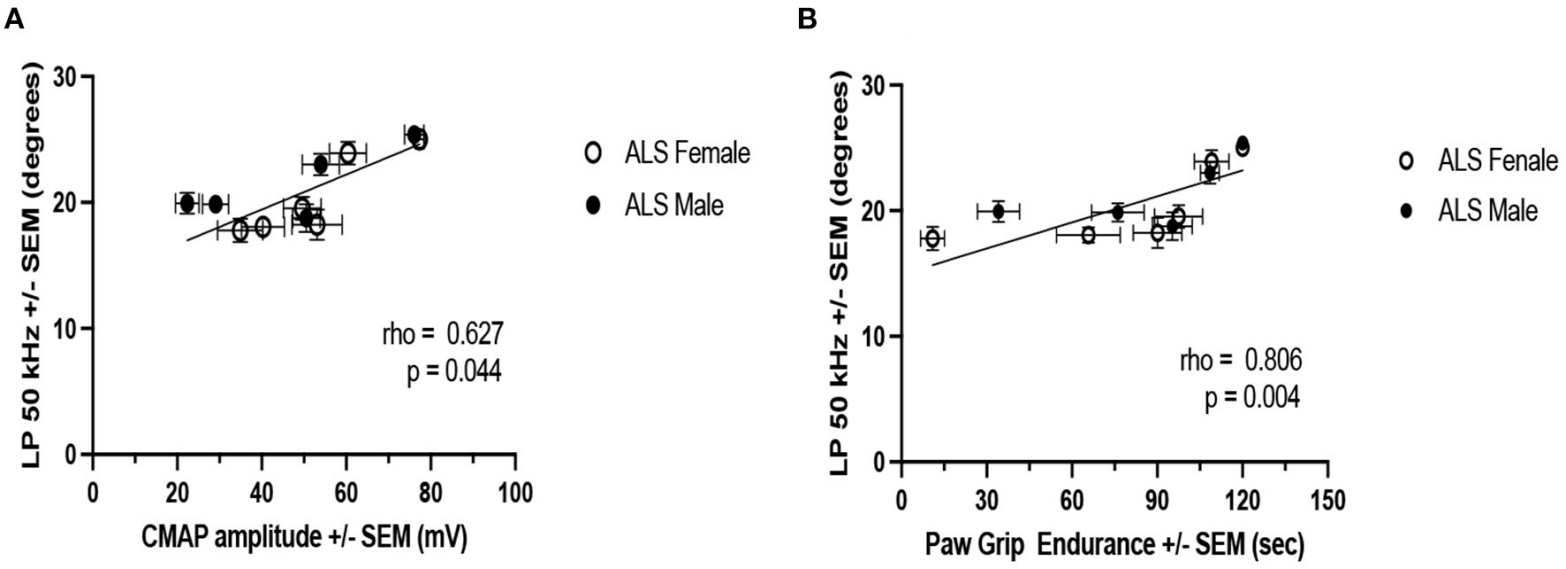
Correlation of LP 50 kHz with PGE and CMAP amplitude. **(A)** PGE. **(B)** CMAP amplitude. For this analysis, weekly data from all ALS animals are averaged by sex.

Figure 6 shows the correlations between LP 50 kHz and PGE and CMAP amplitude. For this analysis, the data from each week was averaged across all the animals by sex. LP 50 kHz correlates with both PGE and CMAP amplitude (Spearman rho: 0.806 and 0.627, respectively; *p*-values: 0.004 and 0.044, respectively) suggesting that these biomarkers could be used interchangeably to follow disease progression in this model of ALS.

### Comparison of disease onset using motor score vs. LP 50 kHz

**Figure 7:**
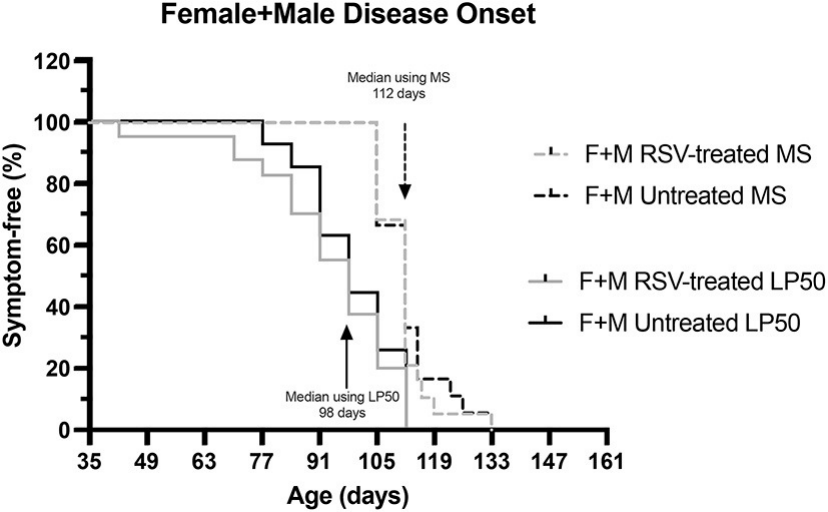
Kaplan-Meier curves comparing disease onset for combined (Female + Male) RSV-treated vs. untreated ALS mice as determined by MS or by LP 50 kHz. Disease onset was determined either as the time of the first recorded MS = 3 or as the time of 20% reduction in LP 50 kHz value from baseline. The median time of disease onset was 112 days for both RSV-treated and untreated animals using MS and 98 days for both RSV-treated and untreated animals using LP 50 kHz as the biomarker.

Finally, we compared the median time of disease determined using the MS with that obtained using LP 50 kHz as the biomarker. Disease onset was determined either as the time of the first recorded MS = 3 or as the time of a 20% reduction in LP 50 Hz value from baseline. Figure 7 shows the resultant Kaplan-Meier curves. The median time of disease onset was 112 days for both RSV-treated and untreated animals using MS and 98 days for both RSV-treated and untreated animals using LP 50 kHz. Although no RSV treatment benefit was detected using either strategy, the median time of disease onset was 14 days earlier using the electrical impedance parameter LP 50 kHz as compared to the MS value.

## Discussion

The results of the present study are 2-fold: (1) Despite evaluating a number of different biomarkers to assess drug efficacy, RSV at the dose and route of administration used here (i.e., 120 mg/kg/day; orally in the chow) showed no beneficial effect in SOD1-G93A ALS mice. As determined by MS analysis, RSV did not delay disease onset or increase overall survival (2). The various biomarkers evaluated showed different abilities to detect disease onset and track disease progression. EIM had the ability to detect disease onset sooner than two other biomarkers evaluated here, i.e., PGE and MS, and was equivalent to the ability of CMAP to detect deviations from baseline values, at ~10 weeks of age.

The lack of efficacy of RSV treatment was unexpected since an earlier study reported a modest therapeutic effect of RSV in this mouse ALS model, albeit that study was conducted using a slightly larger dose of RSV (31). Although our target dose was 160 mg/kg/day, due to pandemic-related delays of more than 6 months, the actual dose of RSV used in the present study was ~25% less than that used previously (31) and could account for the differences seen here. Moreover, whereas the mice in the previous study received RSV in their chow up until the time of their death, in the present study, all ALS mice were switched to Gel-packs, i.e., ending RSV treatment, as soon as the first ALS mice in either group showed signs of paralysis in at least one hindlimb (MS = 1) at ~15–16 weeks of age. This switch was required because mice were not housed individually and once a Gel-pack was provided in the cage for one animal, all other animals in that cage would have access to the Gel-pack as well and could favor it over the RSV-incorporated chow. Of note, some ALS animals then went on to live another 2–3 weeks without receiving any RSV in their chow. This could have had a detrimental impact on their survival, particularly if treatment with RSV is critical at these very late stages of the disease. Another difference between these two studies was the method used to determine the endpoint. A righting reflex (i.e., the time it takes for an animal to right itself after being placed on its side) of >30 s was used in the previous study (31) but could not be used here due to restrictions by our IACUC that were designed to decrease suffering in the mice. Rather, we were required to euthanize mice when their MS reached a value of 0 (i.e., when both hindlimbs were paralyzed thus prohibiting their access to the food in the Gel-packs). Therefore, in the absence of a common endpoint (7), it is difficult to compare the effectiveness of RSV treatment as reported in these two studies.

Nevertheless, we were able to compare the different biomarkers evaluated here for their ability to detect disease onset and track disease progression. As shown in Figure 3, all biomarkers that we examined were able to follow disease progression in this ALS model, although body mass and MS appear especially insensitive to disease status until relatively late in disease progression. Of the remaining biomarkers, LP 50 kHz and CMAP amplitude appear to be the strongest indicators of disease-induced deviation from baseline for several reasons. First, when all data from all ALS mice (i.e., RSV-treated and untreated from both sexes) were combined, LP 50 kHz and CMAP amplitude were able to identify a deviation from their respective baseline values at just 10 weeks of age, earlier than any of the other biomarkers examined. Second, the subsequent decline in LP 50 kHz appeared linear, thus allowing us to determine a rate of disease progression. The results obtained in the present study using EIM as a biomarker are very similar to those that we described previously in this same ALS model when studying the potential therapeutic effects of the drug riluzole (24). Although there was no drug benefit found in either study, EIM was able to track disease onset and progression as well as or better than several other biomarkers in both studies. Finally, there is a good correlation between LP 50 kHz and PGE and CMAP amplitude, indicating that alterations LP 50 kHz values reflect muscle fiber atrophy, decreased muscle strength and deteriorating motor function (18) and could be used interchangeably to follow disease progression.

Critically, EIM appeared to perform similarly to CMAP in these analyses. CMAP is a relatively familiar concept to the clinical neurophysiology community and can be easily obtained with standard nerve conduction equipment, as was the case here. However, it is important to point out that EIM has three valuable characteristics that makes its application in humans especially appealing. First, it does not require the stimulation of nerve. This allows EIM to be performed easily on many different muscles, including even paraspinal muscles, for which a CMAP would be virtually impossible to obtain given the inaccessibility of the nerves. This is especially important in ALS, where tracking muscle deterioration may benefit from not being limited to only the most distal muscles in the upper or lower extremities. Indeed, it has even been used on the tongue successfully (36). Second, it is entirely painless. Third, it is exceedingly fast to perform. These last two features are especially important for human application since it allows application of the technique to be tailored to the region(s) most rapidly progressing in any patient, which can be quite varied in human disease. It also means that it is possible to even perform measurements at home for disease tracking purposes (37). Thus, even if EIM performs similarly to CMAP, in human application, the technique is simply more versatile as tool to monitor deterioration and potential response to therapy.

In addition to the unfortunate reduction in dose of RSV due to the pandemic, limitations to this study include the fact that it was not blinded to the presence or absence of RSV in the chow (since the chow was color-coded); however, this issue would probably only be of concern if a treatment effect had been identified. Second, only a single dose of RSV was studied. Ideally, it would have been preferable to study several other doses of RSV, both higher and lower. Future studies could deliver RSV in drinking water as noted previously (38); however, the limited aqueous solubility of RSV may preclude this approach especially when high daily doses of RSV are required. Alternatively, lower doses of RSV, possibly used in conjunction with other drugs (39) or treatment with other polyphenolic compounds altogether (28) might provide detectable therapeutic benefit in this ALS model. A third limitation of the present study is that we chose to use the PGE test rather than rotarod performance as a measure of muscle health. Whereas, the PGE test, or hanging wire test, is a basic, inexpensive motor test which requires only balance and grip strength and no significant training, the rotarod test is a more complex test requiring balance and motor coordination as well as muscle strength to perform and necessitates special equipment and training of the animals (40). Nevertheless, since rotarod is often used to follow disease progression in ALS mice (1–3, 31), it will be important in future studies to evaluate EIM and rotarod in a side-by-side comparison to determine their respective ability to detect disease onset, track progression and evaluate drug efficacy. A fourth limitation of the present study is that we did not examine any potential targets of RSV in the spinal cord, e.g., sirtuin 1, as described in the previous study (31) and therefore we cannot draw any conclusions regarding the level of RSV in this target tissue. A fifth limitation is that interpretation of the validity of biomarkers using this ALS model is challenging since it is difficult to show biomarker correlation to survival because the mice die over a relatively short period of time, especially when faced with strict mandatory euthanasia criteria. A final limitation of the study is the lack of any standardized commercial surface EIM measurement tool for mice that could be used to replicate our findings. However, to address this issue, we have recently published information about how to create a surface array with an identical footprint to the one used here, as well as specific step-by-step instructions describing and demonstrating how to perform EIM in rodents (34) with the hope of expanding the use of EIM in preclinical studies.

In addition to evaluating the therapeutic efficacy of RSV in model of ALS, another major goal of this study was to determine if surface EIM could be sensitive to a treatment effect in ALS mice. Indeed, although EIM has been studied in a variety of neuromuscular disorders and has been shown to be sensitive to deterioration, there remains a dearth of data establishing its potential capability to detect a beneficial effect of therapy in either humans or animals. In non-motor neuron conditions, a therapeutic effect was identified in boys with Duchenne muscular dystrophy (41) who began corticosteroids and also in humans recovering from disuse atrophy (42). The only example of EIM's ability to detect a potential therapeutic benefit in ALS was in a single patient in a study of intraspinal stem cell implantation who also experienced a clinical improvement (43). Since RSV at the dose given did not produce any clinical effect here, it is at least reassuring that EIM also did not identify one.

In summary, whether RSV has a potential therapeutic effect, remains uncertain and additional study of this nutraceutical at doses of 160 mg/kg/day and higher in mice, in these authors' opinion, remains worthy of study. Unfortunately, it is challenging to engage clinical trialists to pursue a study of RSV since it is a readily available nutraceutical and would be easy for trial participants to supplement on their own. Moreover, its nutraceutical status limits the financial incentive to encourage its use since there is no available patent protection. The second motivation for this study was to assess the technique of EIM, and the data collected here continue to support the technology's use in the assessment of motor neuron disease progression. Further development of a standardized animal system and also a regulatory approved system for human use is currently underway. Once available, EIM can be added to the collection of available tools to assess disease progression in preclinical as well as clinical studies of ALS.

## Data availability statement

The original contributions presented in the study are included in the article/Supplementary material, further inquiries can be directed to the corresponding author.

## Ethics statement

The animal study was reviewed and approved by Beth Israel Deaconess Medical Center Institutional Animal Care and Use Committee.

## Author contributions

SR conceived the experiment(s). JN, CS, and PL conducted the experiment(s). JN, CS, and SR analyzed the results. JN and SR wrote the manuscript. All authors reviewed the manuscript. All authors contributed to the article and approved the submitted version.
