# Supplementary figures and images for: Assessing the therapeutic impact of resveratrol in ALS SOD1-G93A mice with electrical impedance myography
Source: Front Neurol. 2022 Dec 22;13:1059743. doi: 10.3389/fneur.2022.1059743 (PMC9813785; doi:10.3389/fneur.2022.1059743)

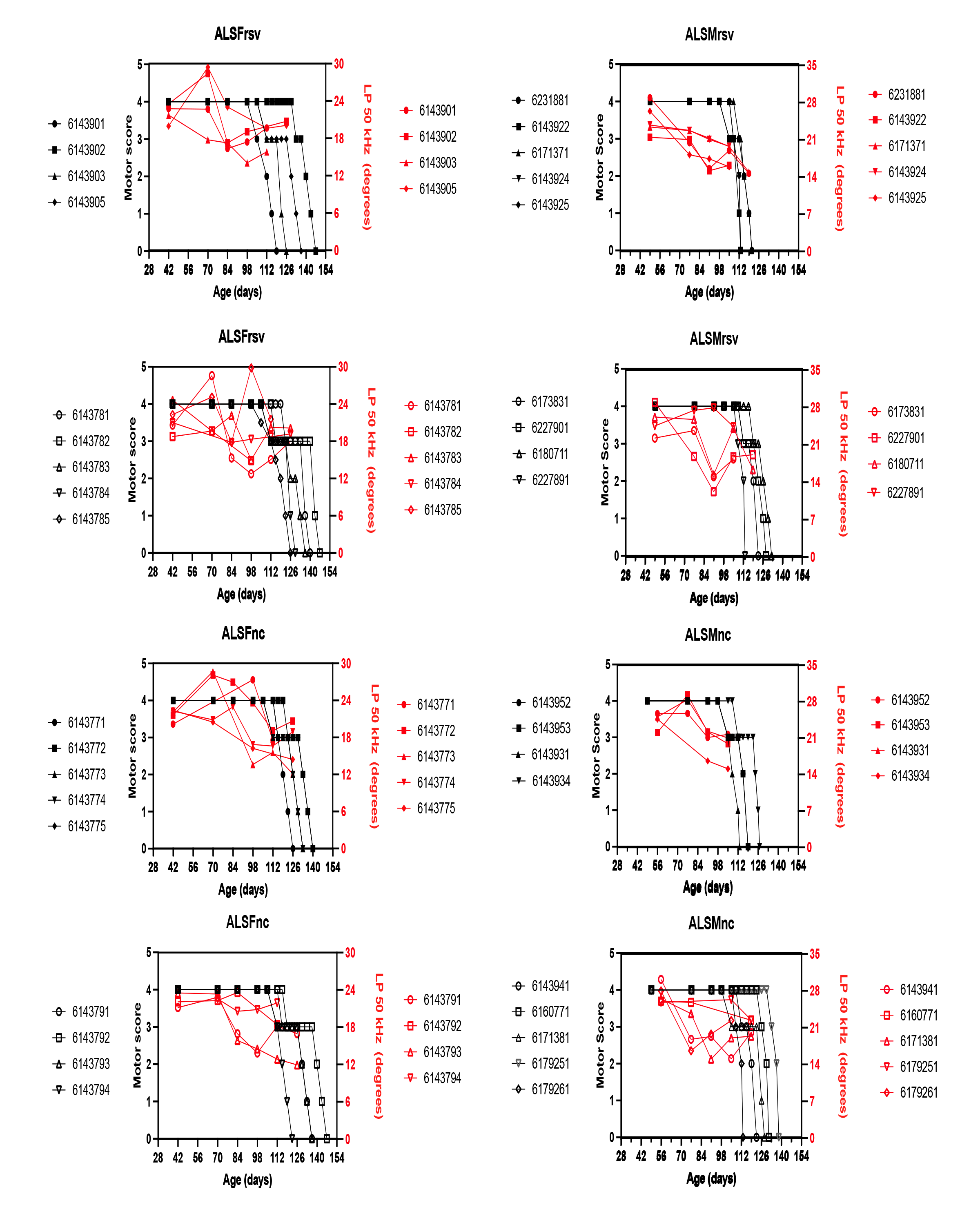

Supplement: Supplementary file 2 [file Image_1.TIF]
